# Assembly and phylogenetic analysis of the mitogenome of *Piophila megastigmata* (Diptera: Piophilidae), a forensically important species

**DOI:** 10.1080/23802359.2022.2107451

**Published:** 2022-08-08

**Authors:** Chengtao Kang, Wang Xu, Yu Wang, Liangliang Li, Yinghui Wang, Yanan Zhang, Jiangfeng Wang

**Affiliations:** Department of Forensic Medicine, Soochow University, Suzhou, China

**Keywords:** Mitogenome, Piophilidae, evolution, necrophagous fly

## Abstract

*Piophila megastigmata* (McAlpine, 1978) (Diptera: Piophilidae) is a forensically important species, but it was not found in China until recently. In this study, the first complete mitogenome of *P. megastigmata* was sequenced using the next-generation sequencing, which was also the first mitogenome of Piophilidae. Our mitochondrial assembly has a length of 15,410 bp, which consists of 22 tRNA genes, 13 protein-coding genes (PCGs), two rRNA genes, and a non-coding control region. This study provides a molecular basis to infer the minimum postmortem interval with *P. megastigmata* and a better understanding of the phylogeny of the order Diptera.

## Introduction

*Piophila megastigmata* (McAlpine, 1978) and *Piophila casei* (Linnaeus, 1758) are the only two species of the genus *Piophila* Fallén, 1810 (Ozerov 2004). *Piophila casei* is a well-known species due to its economic and medical interest, but *P. megastigmata* is mostly an unknown species. In recent years, this species has been collected from human and animal carcasses (Castro et al. 2012), and it can be used to infer the minimum postmortem interval (PMImin) in forensic medicine.

For a long time, *P. megastigmata* was only reported in South Africa (Braack 1986), but it has recently been identified in our forensic investigation in Suzhou, China. Thus, *P. megastigmata* may have a wider distribution than was initially thought. On the other hand, it shares feeding habits and ecological niche with *P. casei* (Martín-Vega 2011), which can lead to misleading identifications due to their similar morphology. Therefore, accurate and reliable identification is urgently required.

Mitogenomes are considered to be ideal for providing phylogenetic relationships (Cameron 2014), while no mitogenomes of the family Piophilidae have been published in Genbank up to now. In this study, we report the first complete mitogenome of *P. megastigmata*, along with an analysis of its gene arrangement, providing a better understanding of the phylogenetic relationships in the order Diptera.

## Materials and methods

### Insects

The *P. megastigmata* specimens used in this study were collected from a pig carcass placed in a field environment in Suzhou, China (31°21′N, 120°53′E) in April 2021, and deposited in the insect specimen room of Laboratory of Forensic Entomology (contact person: Jiang-Feng Wang, email: jfwang@suda.edu.cn) under the voucher number PM-20210423. Species identification was conducted under a Zeiss 2000-C stereomicroscope (Jena, Germany) according to the identification keys provided by Martín-Vega et al. (2011).

### DNA extraction, sequencing, and assembly

Total genomic DNA of *P. megastigmata* was extracted using Rapid Animal Genomic DNA Isolation Kit (Sangon Biotech, China) following the manufacturer′s instructions. 1% agarose gel electrophoresis was used to detect DNA integrity (voltage: 200 V, time: 30 min), and Qubit was used to detect the concentration of the DNA sample.

To obtain the total DNA of *P. megastigmata*, we used DNA Library Prep Kit from Illumina (NEB, USA) for library preparation. Sequencing was constructed on Illumina Hiseq2500 Platform with HiSeq PE150 mode (Paired-end) (Sangon Biotech, China).

Prior to quality control, we used the statistical information of BBtools to evaluate the quality of the original data. After that, the raw data was quality-controlled with BBduk and BLAST+, and the NOVOPlasty software was used for de novo assembly of the mitogenome with the obtained clean reads.

### Annotation and analysis

The genes of the mitogenome were predicted using MITOS2 Server (http://mitos.bioinf.uni-leipzig.de/index.py) (Bernt et al. 2013). Meanwhile, MEGA 7.0 (Kumar et al. 2016) was used to analyze the nucleotide composition. Finally, the circular map of the complete mitogenome was drawn with OGDRAW (Lohse et al. 2013).

We used data from the newly sequenced mitogenome of *P. megastigmata* and those of 10 other taxa for phylogenetic analysis of the order Diptera. As outgroups, we used one species from the order Coleoptera. The bootstrap consensus tree was inferred by the Maximum likelihood (ML) method based on the Kimura 2-parameter model with 1000 bootstrap replicates. All the above alignments, analyses, model selection, and phylogeny reconstruction were performed in MEGA 10.0.

## Results and discussion

### Mitogenome organization and nucleotide composition

After assembly, the mitogenome of *P. megastigmata* is 15,410 bp in length. In addition, two rRNA genes, four PCGs, and seven tRNA genes were located in the light strand, while the other genes were located in the heavy strand. The nucleotide composition was 39.2% A, 37.2% T, 14.1% C, and 9.5% G, showing a biased A + T ratio (76.4%). The gene composition and arrangement of *P. megastigmata* are identical to those of the ancestral insect (Clary and Wolstenholme 1985).

### Protein-coding genes

The majority of PCGs are encoded by the H strand and only *ND1*, *ND4*, *ND4L*, and *ND5* are encoded by the L strand. Three types of start codons – ATT, ATA, and ATG – are used, of which ATG is the most common used start codon. However, the start codon of *COI* gene was not found. Additionally, there are four types of stop codon: TAA, TAG, TA, and T, of which TAA is the most commonly used, while *COII* and *ND4* genes stop with incomplete stop codon TA or T. In general, truncated termination codons are used in metazoan mitogenomes, and are commonly modified to a complete TAA stop codon by post-transcriptional polyadenylation (Nelson et al. 2012).

### Phylogenetic analysis

**Figure 1.**
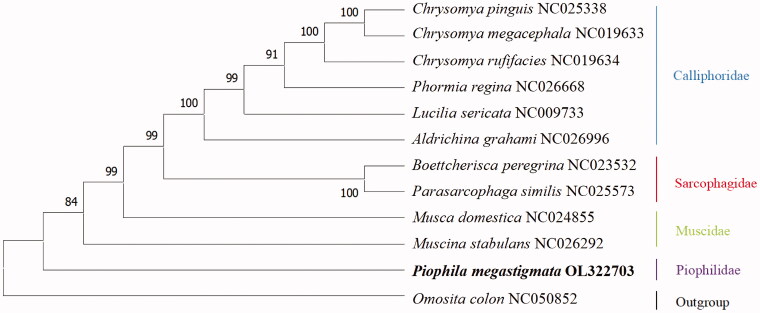
Molecular phylogenetic analysis of order Diptera by maximum likelihood (ML) method based on complete mitogenomes.

The NJ phylogenetic analysis with 11 complete mitogenomes (one generated in this study and 10 obtained from the GenBank) is conducted with MEGA 10.0 (Figure 1). As shown in the figure, the clustering results of each branch are consistent with those of the taxonomy. Five clades were generated in the phylogenetic tree, representing Calliphoridae, Sarcophagidae, Muscidae, Piophilidae, and outgroup, respectively. Phylogenetic analysis results support that *P. megastigmata* is more closely related to the species of family Muscidae.

## Conclusions

Through Illumina DNA sequencing and assembly, the mitogenome sequence of *P. megastigmata* is 15,410 bp in length. In the study, we reported the first complete mitogenome sequencing of *P. megastigmata*. The mitogenome sequence will be an important resource for further molecular studies with *P. megastigmata*.

## Data Availability

Mitogenome data supporting this study are openly available in GenBank at: https://www.ncbi.nlm.nih.gov/nuccore/OL322703. Associated BioProject, SRA, and BioSample accession numbers are https://www.ncbi.nlm.nih.gov/bioproject/PRJNA774910, https://www.ncbi.nlm.nih.gov/sra/SRR16588099, and SAMN22591380, respectively.
